# Osmotic demyelination syndrome and thoughts on its prevention

**DOI:** 10.1007/s40620-021-01081-3

**Published:** 2021-06-05

**Authors:** Philip J. G. M. Voets, Roderick P. P. W. M. Maas, Nils P. J. Vogtländer, Karin A. H. Kaasjager

**Affiliations:** 1 Department of Nephrology, University Medical Centre Utrecht, Heidelberglaan 100, 3584 CX Utrecht, The Netherlands; 2 Department of Neurology, Radboud University Medical Centre, Nijmegen, The Netherlands; 3 Department of Nephrology, Gelre Hospital Apeldoorn, Apeldoorn, The Netherlands

**Keywords:** Hyponatremia, Osmotic demyelination syndrome, Desmopressin clamp, Equation

## Introduction

**Fig. 1:**
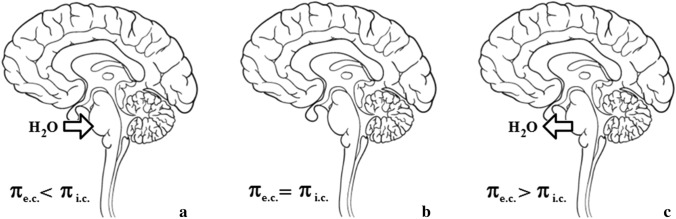
Osmotic water movement in the brain and pathophysiology of osmotic demyelination syndrome. If the effective osmolarity of the extracellular compartment (π_e.c._) is lower than the effective osmolarity of the intracellular compartment (π_i.c._), water moves into the brain cells (**a**). This occurs in hypotonic hyponatremia. If the effective osmolarity of the extracellular compartment subsequently becomes equal to the effective osmolarity of the intracellular compartment, movement of water between both compartments ceases (**b**). This occurs when brain cells have adjusted their intracellular osmolarity to hypotonic hyponatremia by reducing their cytosolic solute content. If the effective osmolarity of the extracellular compartment is higher than the effective osmolarity of the intracellular compartment, water moves out of the brain cells (**c**). This occurs if long-standing hypotonic hyponatremia is corrected. If the water losses in (**c**) are large enough and occur relatively rapidly (> 8 mmol/L/day), massive lysis of glial cells may ensue, leading to osmotic demyelination syndrome (ODS). The arrow in (**a**) points toward the basilar part of the pons, which contains fibers of the corticospinal and corticobulbar tracts and is especially vulnerable to a rapid rise in plasma osmolarity. In addition to central pontine myelinolysis, characterized by spastic tetraparesis, dysarthria, and dysphagia, there is frequently involvement of other central nervous system structures, such as the basal ganglia and thalami (extrapontine myelinolysis) [1]

Osmotic demyelination syndrome (ODS) is a devastating clinical repercussion of the body’s inability to accurately respond to a rapid rise in plasma tonicity. It usually occurs in malnourished individuals with long-standing hyponatremia that was corrected too rapidly (Fig. 1) [1, 2]. Here, after presenting a patient who unfortunately developed ODS, we discuss novel insights into its prevention.

**The case**. A 46-year-old female patient with a medical history of hypertension, for which she used hydrochlorothiazide, presented to the Emergency Department with confusion and lethargy. Over the previous weeks, she had consumed excessive amounts of alcohol, accompanied by poor oral intake. Her general physical examination was unremarkable, except for a heart rate of 110 beats per minute. She was normotensive and weighed 62 kg. Neurological examination revealed disorientation, restless behavior, and a staggering gait, but no paresis or appendicular ataxia. A CT scan excluded structural intracerebral abnormalities. Her plasma sodium concentration turned out to be 95 mmol/L with a measured plasma osmolarity of 198 mOsmol/L. Her plasma potassium concentration was also low at 2.9 mmol/L and she was normoglycemic. Urine osmolarity on admission was 248 mOsmol/L, implying non-osmotic antidiuretic hormone (ADH) release in the context of profound hyponatremia, with a urine sodium concentration of 36 mmol/L. Hypothyroidism and hypocortisolism were ruled out. The internist concluded that her hypotonic hyponatremia was probably caused by chronic hydrochlorothiazide use and poor oral intake and ordered boluses of hypertonic saline (3.0%-NaCl), striving for a maximum correction rate of the plasma sodium concentration of 10 mmol/L in the first 24 h. Her plasma sodium concentration and urine output were strictly monitored (Table 1). Her plasma sodium concentration increased by 5 mmol/L in the first 7 h, which prompted conversion to a 5.0%-glucose infusion. After 6 h of stable plasma sodium concentrations, infusion was switched to 2.5%-glucose/0.45%-NaCl. The correction was then suddenly accompanied by massive diuresis and a decrease in urine osmolarity to 46 mOsmol/L. Seventeen hours after presentation, her plasma sodium concentration was 105 mmol/L. As a rescue strategy, intravenous desmopressin was administered along with 5.0%-glucose infusion. Over the following days, her plasma sodium concentration gradually increased toward normonatremia. A week later, however, she developed severe tetraparesis and respiratory insufficiency requiring orotracheal intubation. A brain MRI scan showed T_2_/FLAIR-hyperintense, T_1_-hypointense signals centrally in the pons, basal ganglia, and thalami, confirming the diagnosis of ODS.

**Table 1:** Course of the patient’s plasma sodium concentration (where (A) refers to a radial artery puncture), urine osmolarity, urine output, and intervention on the day of her admission

Hours since admission	Plasma sodium concentration	Urine osmolarity	Urine output	Intervention
0	95 mmol/L	248 mOsmol/L	–	3.0%-NaCl (bolus of 100 mL)
1	< 100 mmol/L (A)	246 mOsmol/L	125 mL/h	–
2	98 mmol/L	–	100 mL/h	Ringer’s infusate (bolus of 250 mL)
3	98 mmol/L	–	100 mL/h	–
5	100 mmol/L (A)	102 mOsmol/L	200 mL/h	5.0%-glucose (bolus of 1000 mL)
10	102 mmol/L	173 mOsmol/L	–	2.5%-glucose/0.45%-NaCl (? mL)
12	101 mmol/L	252 mOsmol/L	400 mL/h	2.5%-glucose/0.45%-NaCl (? mL)
15	102 mmol/L	152 mOsmol/L	700 mL/h	5.0%-glucose (1000 mL/h)
17	105 mmol/L	46 mOsmol/L	850 mL/h	5.0%-glucose (? mL)/DDAVP
18	102 mmol/L	92 mOsmol/L	1150 mL/h	5.0%-glucose (? mL)/DDAVP
22	100 mmol/L	50 mOsmol/L	700 mL/h	5.0%-glucose (? mL)/DDAVP

It can be seen that an increase in the plasma sodium concentration of 10 mmol/L occurred in the first 17 h after admission, exceeding the maximum allowable correction rate

## Lessons for the clinical nephrologist

Our case demonstrates the dramatic neurological sequelae of an overly rapid correction of profound hypotonic hyponatremia of multifactorial aetiology, which—although anticipated—could not be prevented [1–3]. Here, saline infusion removed the hydrochlorothiazide-induced hypovolemic ADH stimulus, which resulted in a considerable increase in renal free water clearance and a steep rise in plasma sodium concentration. This dangerous phenomenon is commonly referred to as “auto-correction” [4]. It should be noted that the mechanism of thiazide-associated hyponatremia is probably more complex than simple hypovolemia-mediated ADH release and has recently been shown to also involve disrupted prostaglandin E_2_ transport in the renal tubular epithelium [5]. Furthermore, reintroduction of solutes in the form of saline after a prolonged period of inadequate intake strongly increased the patient’s urine output, adding to the auto-correction [4]. Her poor intake may also have contributed to intravascular volume depletion. The depth of the patient’s plasma sodium concentration and her responsiveness on presentation imply that the hypotonic hyponatremia was chronic in nature. Therefore, her pontine cells will have had ample time to adapt to the chronic plasma hypotonicity by decreasing their cytoplasmic solute content, but not enough time to adjust to the rapid rise in plasma sodium concentration when the hypovolemic ADH stimulus was removed. It could be argued that her “malnourished” pontine cells were already less capable of adjusting their intracellular solute content in response to any increase in extracellular tonicity [1]. Hypokalemia has also been described as a risk factor for the development of ODS, probably because it often reflects a poor nutritional status or hypovolemic activation of the renin–angiotensin–aldosterone system, both of which predispose for auto-correction [1]. In our case, however, the observed hypokalemia was most likely the result of chronic hydrochlorothiazide use and malnutrition.

## Proactive desmopressin clamp, the Voets equation and hypertonic saline

In order to forestall neurological complications, guidelines recommend a maximum allowable correction rate of the plasma sodium concentration of 8 to 10 mmol/L in the first 24 h, followed by 8 mmol/L per 24 h over the next days [2, 3]. The maximum allowable correction of extreme hyponatremia should be even slower, since the relative increase in plasma tonicity is larger. A proactive “desmopressin clamp” (PDC) with hypertonic saline boluses is an effective, safe, but relatively unfamiliar treatment strategy for patients with severe hypotonic hyponatremia who are at risk for rapid auto-correction and ODS [6, 7]. PDC, although counter-intuitive at first glance, is intended to control renal free water clearance through the administration of desmopressin, a synthetic ADH analogue. A rational initial dose is 2 µg i.v., after which the following doses depend on urine osmolarity and output [6, 7]. The treating physician can then correct the plasma sodium concentration in a controlled fashion by administering calculated hypertonic saline boluses without being surprised by sudden water diuresis when the endogenous ADH release falls [6, 7]. This proactive strategy is different from a reactive strategy or rescue strategy, as was attempted in the discussed patient [7].

The central problem for physicians when initiating a PDC is to accurately predict the increase in plasma sodium concentration in response to saline infusate to make sure that the correction limit is not exceeded. Many physicians rely on the Adrogue-Madias equation to estimate this change [4, 6, 8]. A major and frequently cited issue with this model is that it solely looks at the redistribution of crystalloid infusate and disregards any subsequent renal water and solute handling. Therefore, calculations according to the Adrogue-Madias equation are short-term predictions, and their accuracy quickly breaks down as time passes [8, 9]. Ignoring renal infusate handling will lead to an imprecise prediction of the “net”—clinically relevant—effect of saline infusion on the plasma sodium concentration. A novel model—hereafter referred to as the Voets equation—has recently been derived and validated for syndrome of inappropriate antidiuretic hormone (SIADH) patients. This model is based on the electrolyte-free water balance that considers both infusate input and renal output under the condition of relatively fixed urine osmolarity. Therefore, it is an ideally suited model to predict the net change in plasma sodium concentration in response to crystalloid infusate boluses with a PDC (dubbed “therapeutic SIADH”) [8, 9]. Because sudden changes in endogenous ADH release are not an issue with a PDC, the patient essentially has a fixed urine osmolarity. For this particular scenario, we believe that the Voets equation—Eq. (1) below—is conceptually better suited than the Adrogue-Madias model, as previously shown for SIADH patients: [8, 9]

1 $$\Delta [Na^{ + } ]_{p} = \frac{{[Na^{ + } ]_{p} V_{i} }}{TBW}\left( {1.7\frac{{o_{i} }}{{o_{u} }} - 1} \right)$$

Here, the parameters $\Delta[Na^+]_p$, $[Na^+]_p$, ${V}_{i}$, $TBW$, ${O}_{i}$, and ${O}_{u}$ represent the predicted change in plasma sodium concentration, initial plasma sodium concentration, infusate volume, total body water, infusate osmolarity (which equals infusate tonicity for crystalloid fluids), and urine osmolarity, respectively [8]. Suppose that a PDC is applied to the previously presented patient with total body water of 31 L, as estimated from her body weight, and that her urine osmolarity is clamped at approximately 250 mOsmol/L, which corresponds to her urine osmolarity on admission. This is desirable as PDC merely sets the stage for a controlled correction of the plasma sodium concentration; it should not in itself induce significant changes in free water clearance. According to the Voets equation, her predicted change in plasma sodium concentration in response to a 0.50L 3.0%-NaCl bolus (osmolarity: 1,026 mOsmol/L) would be 9.1 mmol/L: [8]

2 $$\Delta [{\text{Na}}^{ + } ]_{p} = \frac{95 \cdot 0.5}{{{31}}}\left( {1.7\frac{1026}{{250}} - 1} \right) \approx 9.1$$

By contrast, the modified Adrogue-Madias equation estimates the net effect of a 0.50L 3.0%-NaCl bolus (sodium concentration: 513 mmol/L) on the plasma sodium concentration as follows: [4, 9]

3 $$\Delta [Na^{ + } ]_{p} = \frac{{V_{i} \left( {[Na^{ + } ]_{i} - [Na^{ + } ]_{p} } \right)}}{{TBW{ + }V_{i} }} = \frac{0.5(513 - 95)}{{31 + 0.5}} \approx 6.6$$

Although no prediction model is infallible and a margin of error is inevitable, this relative underestimation of 2.5 mmol/L could encourage physicians to administer larger volumes of saline to the patient than the Voets equation suggests, potentially causing an overly rapid correction of the plasma sodium concentration. In a similar vein, a 3.0%-NaCl bolus of 0.75L, required to increase this patient’s plasma sodium concentration with 10 mmol/L according to the Adrogue-Madias equation, leads to an estimated change of almost 14 mmol/L when the Voets equation is applied.

In our opinion, a PDC with hypertonic saline boluses, calculated according to the Voets equation, is a rational, safe, and effective treatment strategy for hyponatremic patients at risk for auto-correction and ODS. Obviously, frequent measurements of the plasma sodium concentration remain imperative.
